# Modular Titratable Polypills for Personalized Medicine and Simplification of Complex Medication Regimens

**DOI:** 10.1002/adhm.202301101

**Published:** 2023-08-13

**Authors:** Christina Karavasili, Sahab Babaee, Shruti Kutty, Jacqueline N. Chu, Seokkee Min, Nina Fitzgerald, Joshua Morimoto, Nicoletta Inverardi, Giovanni Traverso

**Affiliations:** ^1^ David H. Koch Institute for Integrative Cancer Research and Department of Chemical Engineering Massachusetts Institute of Technology Cambridge MA 02139 USA; ^2^ Division of Gastroenterology Brigham and Women's Hospital Harvard Medical School Boston MA 02115 USA; ^3^ Department of Mechanical Engineering Massachusetts Institute of Technology Cambridge MA 02139 USA; ^4^ Integrated Gastroenterology Consultants N. Chelmsford MA 01863 USA

**Keywords:** cardiovascular disease, complex medication regimen, dose personalization, modular titratable polypill, pill burden

## Abstract

Simplification of complex medication regimens in polypharmacy positively contributes to treatment adherence and cost‐effective improved health outcomes. Even though fixed dose combination (FDC) drug products are the only currently available single dose poly‐pill regimens, the lack of flexibility in dose adjustment of a single drug in the combination limits their efficacy. To fill the existing gap in drug dose personalization and simplification of complex medication regimens commonly encountered in the treatment of cardiovascular disease, tuberculosis, and tapering of corticosteroid therapy, a modular titratable polypill approach that simultaneously addresses both aspects is proposed. The polypill consists of modular units that contain different drugs at incremental or decremental doses to be assembled in a single titratable polypill at the required dose for each drug through a stacking or interlocking process. The variable dose (VD) modular tablets are subjected to quality control tests and found to comply to pharmacopeia's acceptance criteria and requirements specified in the respective drug monographs. A cost‐effectiveness analysis is conducted supporting the VD strategy as cost‐effective compared to the FDC strategy and more effective and less expensive than standard of care. The VD approach stands to enable pill burden reduction, ease of administration, enhancement of treatment adherence, and potential cost‐saving benefits.

## Graphical abstract

The administration of complex medication schemes that are based on multiple dosage forms taken in multiple or variable daily drug doses is challenging as it is associated with low adherence, medication errors, and poor health outcomes. The single pill with variable‐dose combinations approach stands to contribute to pill burden reduction, ease of administration, enhancement of treatment adherence, and cost‐saving benefits.

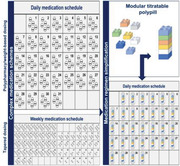

## 1 Introduction

Polypharmacy has been identified as a pressing public health challenge that is encountered within all healthcare settings globally.^[^ ^1^ ^]^ It is often defined as the concurrent use of five or more medications daily and is therefore associated with a higher risk of adverse drug effects, poor health outcomes, and increased healthcare expenditures.^[^ ^2^ ^]^ The prevalence of polypharmacy is expected to experience a surge, as life expectancy has increased worldwide, and a higher proportion of the patient population lives with co‐ and multi‐ morbidities. The problem may intensify as the world undergoes a demographic transition toward an aging population, with the forecasts estimating an increase in those aged 65 years and older from 8% in 2010 to 16% in 2050.^[^ ^3^ ^]^

**Figure 1:**
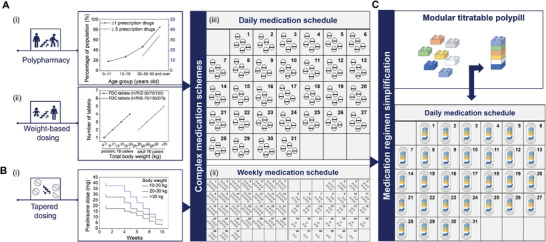
Patterns of prescription drug use among the global patient population that lead to the prevalence of polypharmacy, and formulation interventions to simplify complex medication schemes. A‐a) The rates of concurrent use of one or more and five or more medicines increase with age and are the highest among elderly patients aged 60 years and older. Data are based on a National Health and Nutrition Examination Survey conducted in 2015–2016 in the United States (https://www.cdc.gov/nchs/data/databriefs/db334_tables‐508.pdf#1). b) A weight‐based dosing approach is recommended by the WHO for the treatment of tuberculosis corresponding to an incremental quantity of FDC tablets containing isoniazid (H), rifampicin (R) pyrazinamide (Z) (H/R/Z 50/75/150 mg: pediatric dose during the intensive phase) and isoniazid (H), rifampicin (R) ethambutol (E) (H/R/E 75/150/275 mg: adult dose during the continuation phase).^[^ ^6^, ^31^ ^]^ c) Recommended weight‐based tapering schedule in prednisone dosage during long‐term treatment of pediatric Crohn's disease. B‐a) Polypharmacy results to complex daily medication schemes. b) A gradual decrease of the number of prednisone tablets taken weekly may be required for over 2 months or more during tapering off the medication.^[^ ^7^ ^]^ C) The proposed formulation intervention consisting of a modular titratable polypill may reduce the pill burden and simplify complex medication schedules from multiple pills to a single polypill per day.

According to the National Health and Nutrition Examination Survey conducted in 2015–2016 to monitor prescription drug use in the United States, an age‐related trend was recorded in the use of prescription drugs, which translated to an increase from 18% among children under the age of 12 years to 85% among adults 65 years and older using one or more prescription drugs^[^ ^4^ ^]^ and from 14.5% among adults aged 40–59 to 34.5% among adults aged 60–79 using five or more prescription drugs [**Figure** **1**A(a)].^[^ ^5^ ^]^ Medication regimen complexity may also be associated with disease‐specific guidelines in cases of co‐ and multi‐morbidities in which a weight‐based dosing approach is adopted. One such example is the treatment of pediatric tuberculosis that follows the World Health Organization (WHO) dosing recommendations on the basis of body weight [Figure 1A(b)], requiring the administration of a combination of drugs over several months and which might be further complicated in HIV‐co‐infected children.^[^ ^6^ ^]^ Apart from the number or frequency of medication administered on a daily basis, complexity may also arise when special administration instructions need to be implemented during the course of a treatment. This is highly relevant in the case of tapering off corticosteroid therapy which requires a gradual decrease in the administered drug dose over the course of several weeks or even months [Figure 1A(c)], depending on the initial drug dose and the duration of treatment.^[^ ^7^ ^]^

The administration of complex medication schemes based on multiple dosage forms taken in multiple [Figure 1B(a)] or variable [Figure 1B(b)] daily drug doses is challenging as it is associated with low adherence, medication errors, and poor health outcomes, especially for patients with cognitive impairment, swallowing difficulties, and limited dexterity.^[^ ^8^ ^]^ Medication non‐adherence is a major challenge in polypharmacy, particularly for geriatric patients and patients with multiple co‐morbidities. An inverse correlation has been established between adherence and the medication dosing frequency, with once‐daily dosing resulting in better adherence to twice, three‐ or four‐times daily dosing.^[^ ^9^ ^]^ This is particularly relevant during antihypertensive treatment, especially in low‐ and middle‐income countries that encounter a high burden of hypertension cases.^[^ ^10^ ^]^ Complexity of antihypertensive medication has been identified as a major barrier in achieving consistency in treatment adherence, resulting in suboptimal blood control, and negatively affecting the long‐term management of hypertension.

Simplification of medication regimen in polypharmacy can positively contribute to treatment adherence, further improving health outcomes for the patients and cost‐effectiveness of the healthcare system.^[^ ^11^ ^]^ Several strategies have been adopted in this direction including dose consolidation, the use of long‐acting formulations,^[^ ^12^, ^13^, ^14^, ^15^, ^16^, ^17^ ^]^ or replacing single‐ingredient dosage forms with fixed‐dose combination drug products.^[^ ^8^ ^]^ The polypill concept was first introduced 20 years ago as a preventive strategy to reduce cardiovascular disease.^[^ ^18^ ^]^ The concept, which was based only on meta‐analyses data, entailed a daily treatment with a single six‐component pill that targeted the three risk factors of ischaemic heart disease and stroke (blood pressure, low density lipoprotein cholesterol, and platelet function), claiming that 1 out of 3 people above the age of 55 receiving it would be at a 88% lower risk of heart attacks and an 80% lower risk of strokes. Since then, the initial polypill idea has evolved and been materialized in several clinical trials assessing its efficacy in primary and secondary prevention of cardiovascular disease,^[^ ^19^, ^20^, ^21^ ^]^ its effects on individuals without cardiovascular disease,^[^ ^22^, ^23^ ^]^ as well as the adherence rates among patients at high cardiovascular risk.^[^ ^24^, ^25^, ^26^, ^27^ ^]^ In particular, the randomized, controlled clinical SECURE trial evaluated the effect of a polypill containing aspirin, ramipril, and atorvastatin in 2499 patients over the age of 65 who were diagnosed with a type 1 myocardial infarction (MI) within 6 months prior to study enrolment. Trial findings revealed that treatment with the polypill resulted in significantly higher adherence rates and a lower risk of major adverse cardiovascular events compared to the standard care group, highlighting its potential efficacy as a secondary prevention strategy.^[^ ^21^ ^]^

FDC pills have been the mainstay of treatment for diseases such as tuberculosis, HIV (Combivir‐GlaxoSmithKline: zidovudine and lamivudine), malaria (Coartem‐Novartis: artemether and lumefantrine), and Hepatitis C (Epclusa‐Gilead: velpatasvir and sofosbuvir). Currently, there are several marketed polypill products for the prevention of cardiovascular events (Polycap‐Cadila Pharmaceuticals Limited: atenolol, ramipril, hydrochlorothiazide, simvastatin, and aspirin, CV Pill Kit‐Torrent Pharmaceuticals Ltd: atorvastatin, aspirin, ramipril, and metoprolol, Ramitorva‐Zydus Cadila Healthcare: aspirin, ramipril and atorvastatin). However, dose personalization in all of these cases is solely dependent on the availability of marketed drug products containing the required drug dose for individual patients. Especially for FDC dosage forms, optimal dosing of all drugs might be even more challenging to achieve for the majority of the patient population.^[^ ^28^ ^]^ In particular, geriatric patients who often have multiple co‐morbidities may face significant challenges with FDC medications, as they may require treatment options that are not included in the FDC, or may experience adverse effects that require discontinuation of one of the medications. The potential of employing 3D printing for the manufacturing of personalized polypill formulations has been explored as an alternative to conventional FDCs.^[^ ^29^, ^30^ ^]^ 3D printing technology has the potential to greatly improve the production efficiency of polypills by reducing production steps, enabling rapid manufacturing, and facilitating continuous production. Yet, the requirements for special equipment and training of the pharmaceutical personnel, along with the need to define a new regulatory framework on the use of 3D printing technologies, may hinder their implementation in drug manufacturing in the near future.

To fill the existing gap in drug dose personalization and simplification of complex medication regimens, we propose a modular titratable polypill approach that addresses both aspects at the same time, shown in Figure 1C. The polypill consists of modular units that contain different drugs at incremental doses assembled in a single polypill at the required dose for each individual drug. The single pill with variable‐dose combinations approach stands to contribute to pill burden reduction, ease of administration, enhancement of treatment adherence, and cost‐saving benefits. The adoption of well‐established manufacturing techniques and widely accepted dosage forms from the patient population will facilitate the seamless integration of the modular titratable polypill approach into the existing healthcare systems and the transition from a mass to an individualized treatment approach.

## 2 Results

### 2.1 Designs and Assembly of the Modular Titratable Tablets

**Figure 2:**
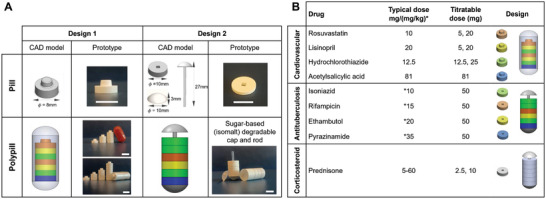
Design and assembly of the modular titratable pills for simplifying complex (cardiovascular, antituberculosis) or tapered (corticosteroid) medication schemes. A) Design 1 corresponds to a dome/dimple‐shaped tablet assembled to a polypill through an interlocking process. The polypill may fit in an AA capsule (up to five tablets) or a 000 capsule (up to 8 tablets). Design 2 corresponds to an annular disk‐shaped tablet assembled to a polypill through a stacking process through a central rod that is secured with a cap. The polypill may fit up to ten tablets. Scale bars in all photos correspond to 1 cm. B) A combination of four drugs used to treat hypertension (rosuvastatin, lisinopril, hydrochlorothiazide, acetylsalicylic acid) or tuberculosis (isoniazid, rifampicin, ethambutol, pyrazinamide) and a corticosteroid (prednisone) were formulated in titratable doses that enable incremental or decremental dose adjustment based on the typically administered drug doses.

Two modular designs were developed to facilitate both instant dose titration and administration of more than one drug in a single polypill, shown in **Figure** **2**A. Design 1 is a stackable modular disk‐shaped tablet (8 mm diameter) composed of a dimple‐shaped protrusion on the top and the same shape cavity on the bottom designed to interlock the tablets by applying mild pressure (Figure S1A, Supporting Information). Assembly of up to 5 and 8 of those tablets will form a polypill that could fit in a standard AA (closed length: 17.5 ± 0.3 mm, diameter: 9.39 ± 0.06 mm) and 000 (closed length: 26.1 ± 0.3 mm, diameter: 9.55 mm) capsule, respectively. Design 2 is an annular disk‐shaped tablet (10 mm diameter) assembled to a polypill via a stacking process through a central rod that is secured with a cap (Figure S1B, Supporting Information). The polypill of this modular design will accommodate up to ten tablets.

Drug selection for the formulation of the modular titratable polypill was based on diseases that are linked to polypharmacy, with the aim to reduce the pill burden and simplify complex medication schedules from multiple pills to a single polypill per day. This is highly relevant in the case of cardiovascular diseases and tuberculosis, which require the administration of five or more individual dosage forms usually more than once per day, as well as conditions that require dose tapering, such as during corticosteroid therapy. Each drug was formulated in titratable doses that enable either incremental dose adjustment based on the typically administered drug dose for the treatment of cardiovascular diseases;^[^ ^32^ ^]^ namely, rosuvastatin, lisinopril, hydrochlorothiazide, and acetyl salicylic acid, and the recommended daily doses of first line antituberculosis drugs for children;^[^ ^33^ ^]^ namely, isoniazid, rifampicin, ethambutol, and pyrazinamide or decremental dose adjustment during prednisone tapering,^[^ ^7^ ^]^ as illustrated in Figure 2B.

### 2.2 Manufacturing of the Modular Titratable Tablets and Quality Control Assessment

**Figure 3:**
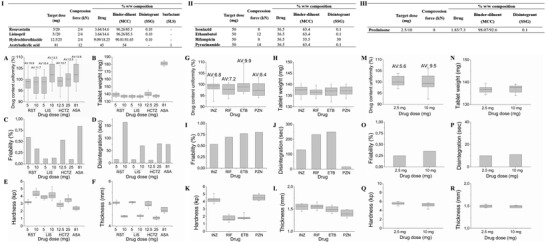
Excipient composition and quality control assessment of the modular tablets. The tables show the target dose (mg), compression force (kN) and excipient composition (% w/w) of the modular tablets containing the cardiovascular drugs (I), antituberculosis drugs (II) and prednisone (III). Quality control tests were performed to assess A,G,M) drug content uniformity (*n* = 10, ± S.D.), B,H,N) weight variation (*n* = 20, ± S.D.), C,I,O) friability, D,J,P) disintegration (*n* = 6), E,K,Q) hardness (*n* = 10, ± S.D.), and F,L,R) the thickness of the modular tablets containing the A–F) cardiovascular drugs, G–L) antituberculosis drugs, and M–R) prednisone.

The excipient composition of the different drug formulations and the required force applied for the manufacturing of the modular titratable tablets by direct compression were optimized based on preliminary experiments, shown in **Figure** **3**(I–III). Design 1 was selected for the manufacturing of the tablets containing the cardiovascular drugs and design 2 for the antituberculosis drugs and prednisone. Microcrystalline cellulose (MCC) was used as the binder‐diluent due to high binding capacity even at low compression pressure, generating tablets that are both stiff and stable, yet showing rapid disintegration. Sodium starch glycolate (SSG) was therefore used as the super‐disintegrant at a minimum concentration (0.1% w/w) in most formulations and where necessary, to further promote disintegration of the immediate release dosage forms. The addition of sodium lauryl sulfate (SLS) was found to be critical only in enhancing the dissolution performance of the acetyl salicylic acid tablets. The target weight was set to 137 mg for most formulations to enable the fabrication of tablets with acceptable properties, yet of minimum thickness so that five or more could be assembled in a single polypill. Acetyl salicylic acid required a higher binder concentration for the fabrication of tablets that comply with the pharmacopeia's acceptance criteria and target weight was therefore set to 180 mg.

We started by performing quality control assessments of the individual tablets including drug content uniformity, weight variation, friability, disintegration, hardness, and thickness uniformity tests. The results of the quality control tests of the modular tablets were reported for the cardiovascular drugs in Figure 3A–F, the antituberculosis drugs in Figure 3G–L, and prednisone in Figure 3M–R. According to the content uniformity test, which sets the limits of drug content variance within each tablet, all modular tablet dosage forms were found to comply with the limits specified in the respective drug monographs of United States Pharmacopeia (USP) 34—NF 29 (Figure 3A,G,M). The acceptance values (AV) calculated for the content uniformity test were found to be less than the limit (less than 15.0) in all cases. Tablet weight variation is among the factors that may contribute to variability in content uniformity in tablets. Based on the USP limits for the weight variation test of uncoated tablets with an average weight ranging between 130 and 324 mg, the percentage weight deviation should not exceed 7.5%, which was the case for all drug modular tablet dosage forms fabricated in the current study (Figure 3B,H,N).

The measure of the modular tablets’ resistance to abrasion or fracture was expressed as percentage loss in weight, which according to the USP specification must not exceed 1% of the original tablet weight. None of the modular tablets showed signs of breakage during the course of the test, and friability loss ranged between 0.09% and 0.83% (Figure 3C,I,O). The modular tablets were also subjected to the disintegration time test in water at 37 °C ± 2 ^°^C. Disintegration has been partially correlated to drug dissolution and is affected by several factors, including the nature of the drug and the compression force applied during tablet manufacturing. All tablets disintegrated within less than 15 min meeting the pharmacopeial requirement for uncoated tablets (Figure 3D,J,P), with ethambutol modular tablets showing the longest disintegration time of 4 min and 16 s. The isomaltose‐casted rod and cap that were fabricated for the assembly of the annular disk‐shaped tablets, showed a fast dissolution within 1 and 3 min, respectively.

The assessment of the crushing strength (Figure 3E,K,Q) and thickness (Figure 3F,L,R) of the modular tablets were among the quality control tests performed, that are not included in official compendia and do not have definite limits and acceptance criteria. Both tablet hardness and thickness are affected by the compression force used during their manufacturing and hardness, in turn, can affect both disintegration and dissolution. The average hardness values for the modular tablets ranged between 1.75 ± 0.36 kp for the rifampicin annular disk tablets and 5.54 ± 0.28 kp for the prednisone (2.5 mg dose) annular disk tablets. Even though both drugs were compressed into annular disk tablets using the same force (8 kN), the higher drug content and disintegrant concentration in the rifampicin tablets resulted in dosage forms of significantly lower hardness, compared to the prednisone ones (P<0.05). The thickness of the dome/dimple modular tablets ranged between 4.5 and 5 mm and an inverse correlation between tablet thickness and compression force was observed. On the other hand, all annular disk tablets compressed at 8–14 kN demonstrated similar thickness of ≈1.5 mm.

### 2.3 Dissolution Testing

**Figure 4:**
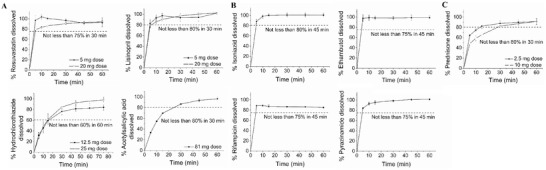
In vitro dissolution profiles of the A) cardiovascular drugs including rosuvastatin, lisinopril, hydrochlorothiazide, and acetylsalicylic acid, B) antituberculosis drug including isoniazid, ethambutol, rifampicin, and pyrazinamide; and C) prednisone from the modular titratable tablets. Dissolution studies were performed under the conditions specified in the individual drug monographs (Table S1, Supporting Information) at 37 °C ± 0.1 °C. Horizontal dashed lines correspond to the dissolution limits (minimum amount of drug dissolved (%) within a specified time point) as defined in the respective drug monographs. Data are presented as mean ± standard deviation (*n* = 4).

In vitro dissolution studies were carried out according to the conditions described in the individual drug monographs (Table S1, Supporting Information). The dissolution profiles of the cardiovascular drugs [**Figure** **4**A(a–d)], antituberculosis drugs [Figure 4B(a–d)], and prednisone (Figure 4C) for the modular tablets were plotted as the amount of drug dissolved (%) versus time. The USP dissolution requirements for each individual drug are summarized in Table S1, Supporting Information.

Notably, all modular tablet dosage forms for the cardiovascular drugs, antituberculosis drugs, and prednisone passed the USP acceptance criteria for drug dissolution. In particular, 5 and 20 mg rosuvastatin modular tablets achieved 96.80% ± 2.78% and 90.46% ± 2.08% drug dissolution, respectively within 30 min [not less than (NLT) 75% in 30 min]. 5 and 20 mg lisinopril modular tablets achieved 93.68% ± 7.14% and 96.95% ± 3.71% drug dissolution, respectively within 30 min (NLT 80% in 30 min). 12.5 and 25 mg hydrochlorothiazide modular tablets achieved 82.53% ± 5.90% and 95.64% ± 4.25% drug dissolution, respectively, within 60 min (NLT 60% in 60 min). 81 mg acetyl salicylic acid modular tablets achieved 86.74% ± 1.47% drug dissolution within 30 min (NLT 80% in 30 min). 50 mg isoniazid modular tablets achieved 100.62% ± 3.54% drug dissolution within 45 min (NLT 80% in 45 min). 50 mg isoniazid modular tablets achieved 98.76% ± 4.90% drug dissolution within 45 min (NLT 75% in 45 min). 50 mg rifampicin modular tablets achieved 86.28% ± 0.95% drug dissolution within 45 min (NLT 75% in 45 min). 50 mg pyrazinamide modular tablets achieved 100.89% ± 1.79% drug dissolution within 45 min (NLT 75% in 45 min). 2.5 mg and 10 mg prednisone modular tablets achieved 87.56% ± 5.110% and 82.22% ± 1.70% drug dissolution, respectively within 60 min (NLT 80% in 30 min).

### 2.4 Cost‐Effectiveness Analysis of the VD Polypill in the Prevention of Cardiovascular Disease

**Figure 5:**
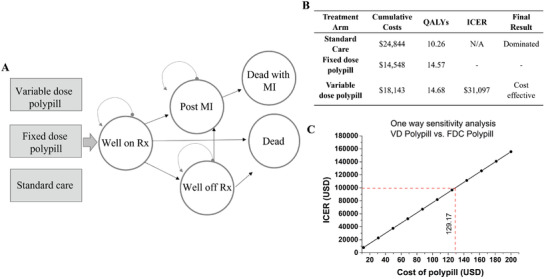
A) Health state transition diagram showing the three treatment arms of standard care, FDC and VD polypill, and the health states in the Markov Cohort model. B) Base case results for the cost effectiveness analysis comparing standard care, FDC, and VD strategies. Standard Care treatment was the most expensive and least effective (dominated). VD is cost‐effective compared to FDC. All results are represented in 2019 US$ with 3% discounting applied. C) One‐way sensitivity analysis showing the VD polypill remains cost‐effective up to a monthly cost of $129.

We then performed cost‐effectiveness analysis to identify whether the polypill strategy would result in a lower pharmaceutical expenditure compared to standard care in the prevention of cardiovascular disease. To achieve that we used a Markov model, and we compared the VD to the FDC polypill strategy and standard care. In the base case analysis, the VD strategy was cost‐effective compared to the FDC strategy with an incremental cost effectiveness ratio (ICER) of $31 097 per quality adjusted life year (QALY). The standard care treatment strategy was the effective and more expensive (dominated) compared to the other two strategies. Base case results are summarized in **Figure** **5**B. One‐way sensitivity analyses were performed to explore the impact of variation in model parameters on the primary outcome. The parameters that changed the cost effectiveness of the VD strategy included higher probability of side effects from the VD polypill (worse than 68%), higher cost of the VD pill (above $129 per month), higher MI probability for VD patients who continued medication after experiencing side effects per year (>1.4%) and higher MI probability for VD patients who experienced no side effects (>1.1%) per year. All sensitivity analyses are summarized in Figures S2–S4, Supporting Information. This preliminary cost‐effectiveness analysis suggests that the VD strategy is cost‐effective compared to the FDC strategy and is also more effective and less expensive than standard care. The VD strategy continued to be cost‐effective up to a cost of $129/month for the polypill. Our study also noted the importance of reducing side effects, as increasing side effects of the polypill limited its cost‐effectiveness. Since the VD polypill is personalized, it is less likely to cause side effects compared to the FDC polypill, which has been one concern limiting the FDC polypill's adoption to date.

## 3 Discussion

FDC drug products are the only currently available solution to address the untenable pill burden and complex medication schemes encountered by polypharmacy patients, especially during lifelong treatment. The rationality of their use, however, is compromised by the fact that dosage adjustment of an individual drug in the combination is not possible without changing the dosage of the other drugs. In this work, we aimed to address this issue through a modular titratable polypill that enables dose adjustment of each individual drug in the combination in order to meet the therapeutic requirements of an individual patient or a defined patient population group. To achieve that, we introduced the element of modularity in conventional tablets that could be assembled in a single polypill through an interlocking or stacking approach. We manufactured two tablet designs that can accommodate in a single polypill incremental doses of up to four drugs, that are commonly used in the treatment of cardiovascular disease (rosuvastatin, lisinopril, hydrochlorothiazide, acetylsalicylic acid) and tuberculosis (isoniazid, rifampicin, ethambutol, pyrazinamide) or decremental drug doses of a corticosteroid, which is commonly required during tapered dosing of prednisone. The modular tablets were subjected to quality control tests and were found to comply to the pharmacopeia's acceptance criteria for disintegration, weight uniformity, and friability, as well as to the drug content and dissolution requirements specified in the respective drug monographs.

In order to get a better understanding on the viability of our titratable polypill in the landscape of therapeutic interventions adopted in polypharmacy, we performed a cost‐effectiveness analysis comparing the costs and health benefits of standard of care and FDCs to our approach. As with all models, the cost effectiveness model was limited by currently available data and certain assumptions made through the analysis. Assumptions in this model included limiting patients to discontinuing medication only due to side effects or non/low adherence, assuming probabilities of a MI for low and non‐adherent patients was the same, all post‐MI patients adhere to their medications fully, and finally, assuming that a side effect would not impact the probability of having a MI. There were also a few limitations to the model introduced by the data used to support it. First, a limitation was that some of the clinical trials used for the model included patients of various ages below and above 60, whereas the model focused on patients at 60 years of age only. Additionally, while the cohort in this model is assumed to not have had a prior MI, some clinical trials used included patients with histories of MI. Therefore, the patient population utilized from the clinical trials did not provide as a complete match to the population studied within our model. Second, a limitation was that the model went on for a lifetime horizon however the clinical trials used to support this model were short term with the longest being 5 years. Third, while most trials administered the same classes of medications as the VD polypill in their treatment arms to patients (combination of 1 blood thinner, 2 anti‐hypertensives, and 1 anti‐cholesterol), the medications themselves were not the same compared to the ones showcased in this paper for the VD polypill. Finally, since the VD polypill, has never been used clinically, the data used for the VD polypill was modeled based on published data from available FDC polypills and standard care pills. The VD polypill was also just modeled for the cardiovascular application, hence, these results do not apply to the TB or the prednisone applications. An updated analysis should be performed if/when clinical data becomes available to understand better whether the VD polypill would make a difference in patient's lives and prove to be cost effective. Therefore, the results shown here should be taken as preliminary only with the intention to improve upon once newer and more significant data is available to use. Overall, we expect that the modular titratable polypill could provide a straightforward path from mass to personalized treatment, leveraging the assets of a well‐established manufacturing technique and patient acceptability of this dosage form.

## 4 Experimental Section

### Materials

Acetylsalicylic acid (ASA, ≥99.0%), cellulose (microcrystalline, powder, MCC), and hydrochloric acid (37%) were purchased from Sigma Aldrich Co. (St. Louis, MO, USA), rosuvastatin calcium salt (RST, ≥98%) and lisinopril dihydrate (LIS, ≥98%) were purchased from GLSyntech Dependable Chemistry (Hatfield, PA, USA), (S,S)‐*N*,*N*'‐*Bis*(1‐hydroxy‐2‐butyl)ethylenediamine dihydrochloride (ethambutol‐ETB, >98.0%) and isonicotinic acid hydrazide (isoniazid‐INZ >98.0%) were purchased from TCI‐Tokyo Chemical Industry Co LTD. (Tokyo, Japan), pyrazinamide (PZN, 99%) was purchased from Thermo Scientific (Waltham, MA, USA), hydrochlorothiazide (HCTZ, 98%) was purchased from BeanTown Chemical (Hudson, NH, USA), sodium lauryl sulfate (SLS, ≥95%) was purchased from J. T. Baker (PA, USA) and SSG (type A NF) was purchased from Spectrum chemical MFG Corp. (New Brunswick, NJ, USA). Milli‐Q water was used in all experimental procedures. The Form 2 printer and the Clear resin for 3D printing were purchased from Formlabs Inc. (Somerville, MA, USA), centrifuge was purchased from Eppendorf (Hamburg, Germany), 2‐part dental grade silicone mix kit (Elite Double 32) was purchased from Zhermack (Badia Polesine RO, Italy), sample cold‐mounting cups were purchased from LECO Corp (St. Joseph, MI, USA), isomaltose (crystalline powder, food grade) was purchased from Amazon.com Inc. (Seattle, WA, USA), and SolidWorks Computer‐Aided‐Design (CAD) software package was purchased from Dassault Systemes (Vélizy‐Villacoublay, France).

### Formulation and Manufacturing of the Modular Tablets

The excipient composition of the different drug formulations is summarized in Figure 3(I–III). The powder mixtures were blended in a SpeedMixer (DAC 150.1 FVZ‐K, SpeedMixer.co.uk, Buckinghamshire, UK) for two consecutive cycles of 3500 rpm for 15 s each. The blended mixtures were then directly compressed on a bench‐top hydraulic single‐punch tablet press (NP‐RD10A, NATOLI, MO, USA) at a target weight of 137 mg for all formulations, except for ASA which was compressed at a target weight of 180 mg, and at compression forces ranging between 2 to 14 kN (Figure 3(I–III). The die fill depth was appropriately adjusted for the different drug formulations to achieve the target weight value. Two different types of punches were used to generate the dome‐dimple shaped and the annular disk modular tablets. The detailed CAD models of the polypill prototypes are shown in Figure S1, Supporting Information. The dome‐dimple shaped tablets were compressed using 8 mm flat punches with a domed lower punch (diameter: 4 mm, height: 2 mm) and a dimpled upper punch (diameter: 4 mm, depth: 2 mm), whereas the annular disk tablets were compressed using 10 mm round flat punches with the upper punch having a central 2 mm hole and the lower punch having a protruding cubic rod (diameter: 2 mm).

### Fabrication of the Dissolvable Parts for the Assembly of the Annular Disk‐Shaped Tablets

The dissolvable components of the Design 2 polypill were designed in CAD (SolidWorks), modified with sprues, and printed on the Form 2 printer using the Clear resin. These positives were then attached to the LECO cold‐mounting sample cups, which were then filled with the mixed 2‐part cure silicone resin to create the casting molds. The isomalt was boiled until molten and poured into the silicone molds, which were then centrifuged in order to remove any air porosity defects in the cast. The cast parts were then removed from the silicone molds and trimmed to design (Figure S1C, Supporting Information).

### Instrumentation and Analytical Methods

HPLC analyses of ethambutol, pyrazinamide, isoniazid, lisinopril, hydrochlorothiazide, acetylsalicylic acid, rosuvastatin, and prednisone were performed on an Agilent 1260 Infinity II HPLC equipped with a diode‐array detector (DAD), quaternary pump, autosampler, thermostat and control module (Agilent Technologies, Santa Clara, CA, USA). Separations with 50 mm Poroshell 120 EC‐C18 columns used a 5 mm guard column of the same stationary phase. Data processing of the output signal was performed using ChemStation software. The specific chromatographic parameters for each analyte are specified in the following section.

Ethambutol analyses were adapted from a quantitative spectrophotometric method using complexation with copper (II).^[^ ^34^ ^]^ HPLC was performed using an Agilent Poroshell 120 EC‐C18 column (3.0 × 50 mm, 2.7 µm) held at 25 °C. A mobile phase of 1 mm copper (II) sulfate and 10 mm ammonium formate adjusted to pH 4.6 with ammonia (A) and methanol (B) was used. Samples were injected at a volume of 10 µL, with an isocratic separation using 7% B at a flow rate of 0.8 mL min^−1^ and 2 min run time. The copper‐ethambutol complex eluted at 1.3 min, and absorbance was measured by the DAD at 280 nm with a bandwidth of 4 nm at a scan rate of 5 Hz.

HPLC of pyrazinamide was performed using an Agilent Zorbax Eclipse XDB C18 column (4.6 × 150 mm, 5 µm) at 35 °C. A mobile phase of water (A) and acetonitrile (B) was used at a flow rate of 1 mL min^−1^. The gradient elution began at 5% B, increasing to 95% B over 4 min, with a stop time of 4.5 min and re‐equilibration time of 1 min. The injection volume was 10 µL. The DAD parameters were as follows: UV wavelength of 244 nm, bandwidth of 4.0 nm, and a scan rate of 5 Hz. The analyte peak eluted at 2.7 min.

HPLC of isoniazid was performed using an Agilent Poroshell 120 Bonus‐RP column (3.0 × 100 mm, 2.7 µm). Separations were performed at a constant temperature of 35 °C. The mobile phase consisted of 0.05% acetic acid in water (v/v) (A) and methanol (B), flowing at 1 mL min^−1^. The gradient program was: 0 min, 0% B; 0.5 min, 0% B; 3 min, 60% B; 3.1 min, 95% B; with a runtime of 7 min and a re‐equilibration time of 3 min. The injection volume was 10 µL. Absorbance was measured at a wavelength of 264 nm and bandwidth of 4 nm at a scan rate of 20 Hz. The analyte peak eluted at 0.7 min.

HPLC of lisinopril was performed using an Agilent Zorbax Eclipse XDB C18 column (4.6 × 150 mm, 5 µm) held at 45 °C. A mobile phase of 20 mm dipotassium phosphate in water at pH 3.00 (v/v) (A) and methanol (B) was used. The isocratic elution consisted of 55% A and 45% B and ran at a flow rate of 1 mL min^−1^ for a total run time of 6 min. The injection volume was 5 µL. The DAD parameters were as follows: UV wavelength of 210 nm, bandwidth of 4 nm, and scan rate of 10 Hz. The analyte peak eluted at 2.0 min.

HPLC of hydrochlorothiazide was performed using an Agilent Poroshell 120 EC‐C18 column (3.0 × 50 mm, 2.7 µm) held at 30 °C. The mobile phase consisted of 0.1% formic acid in water (v/v) (A) and acetonitrile (B), flowing at 1 mL min^−1^. The gradient program was: 0 min, 5% B; 2 min, 30% B; 2.10 min, 90% B; with a runtime of 3.5 min with a re‐equilibration time of 1.5 min. The injection volume was 10 µL. The DAD parameters were as follows: UV wavelength of 272 nm, bandwidth of 4 nm, and a scan rate of 5 Hz. The analyte peak eluted at 1.7 min.

HPLC of acetylsalicylic acid was performed using an Agilent Poroshell 120 EC‐C18 column (3.0 × 50 mm, 2.7 µm) at a constant temperature of 30 °C. A mobile phase of 0.1% formic acid in water (v/v) (A) and acetonitrile (B) flowing at 0.75 mL min^−1^ was used. The gradient program was: 0 min, 5% B; 1 min, 15% B; 4 min, 85% B; 4.5 min, 95% B. The total run time was 5 min with a re‐equilibration time of 2 min. The injection volume was 10 µL. The DAD parameters were as follows: UV wavelengths of 228, bandwidth of 4 nm, and a scan rate of 10 Hz. The acetylsalicylic acid peak eluted at 3.3 min.

HPLC of rosuvastatin was performed using an Agilent Poroshell 120 EC‐C18 column (4.0 × 50 mm, 2.7 µm) held at 35 °C. The mobile phase of 0.1% formic acid in water (v/v) (A) and methanol (B) flowed at a rate of 1 mL min^−1^ at an isocratic composition of 55% B for 8 min. The injection volume was 20 µL. The DAD parameters were as follows: UV wavelength of 243 nm, bandwidth of 4 nm, and a scan rate of 5 Hz. The analyte peak eluted at 2.8 min.

HPLC of prednisone was performed using an Agilent Poroshell 120 EC‐C18 column (4.6 × 50 mm, 2.7 µm) at a constant temperature of 40 °C. A mobile phase of water (A) and acetonitrile (B) was used at a flow rate of 1 mL min^−1^ with an isocratic composition of 40% B and runtime of 6 min. The injection volume was 5 µL. The DAD parameters were as follows: UV wavelengths of 254 nm, bandwidth of 4 nm, and a scan rate of 10 Hz. The analyte peak eluted at 1.0 min.

Rifampicin was quantified at 475 nm on a microplate reader (Infinite 200 PRO, Tecan Trading AG, Switzerland).

### Drug Content Uniformity‐Weight Variation

The drug content uniformity assay was performed in ten randomly selected tablets from each formulation in order to determine whether the individual drug content was within the limits set in the respective drug monographs. Drug quantification was performed according to the analytical procedures described in the Instrumentation and Analytical Methods section. The acceptance value (AV) was calculated for each formulation based on the following formula:

(1) $$\left|M-\bar{X}\right|+ks$$

where *M* is the reference value, *X̄* is the mean of individual drug contents expressed as the percentage of the theoretical drug content, *k* is the acceptability constant (*k* = 2.4 for *n* = 10 units tested) and *s* is the sample standard deviation. Based on the mean value of individual drug contents, *M* = *X̄* if 98.5% ≤ *X̄* ≤ 101.5%, *M* = 98.5% if *X̄* < 98.5% and *M* = 101.5% if *X̄* > 101.5%. The maximum allowed AV was defined to be 15.0.

Weight variation was calculated after weighing 20 randomly selected tablets from each formulation. The weight variation limits for tablets weighing between 130 and 324 mg indicate that the percentage variation should not exceed 7.5.

### Friability

Tablet resistance to abrasion was determined using a friability tester (FT2, SOTAX Corporation, Westborough, MA, USA). A sample of tablets from each formulation (weighing as near as possible to 6.5 g as specified for tablets with a unit mass that equals or was less than 650 mg) was carefully dedusted and accurately weighed. The tablets were placed in the drum and the test was performed at a rotational speed of 25 ± 1 rpm for 4 min. At the end of the test, tablets were dedusted and accurately weighed and the percentage mass loss was calculated. The test was run once if no tablet breakage occurred, and maximum mass loss did not exceed 1%.

### Disintegration

Tablet disintegration was assessed in water at 37 °C ± 2 °C using a disintegration apparatus (DT2, SOTAX Corporation, Westborough, MA, USA). Six tablets from each formulation were randomly selected and placed in each tube of the basket and the time required for complete disintegration of all samples to occur was recorded. The test was run once if all dosage units disintegrated within 15 min. The same procedure was followed to record the time required for the cap/rod parts of the Design 2 polypill to fully dissolve.

### Hardness and Tablet Thickness

The breaking force of the tablets was measured using a hardness tester (MT50, SOTAX Corporation, Westborough, MA, USA). Ten tablets were randomly selected from each formulation and the maximum force required to crush each tablet was recorded at a test speed of 3 mm s^−1^. The thickness of the tablets was measured with an electronic micrometer.

### In Vitro Dissolution Studies

Dissolution studies of the different drug formulations were performed at 37 °C (Vision G2 Elite 8, Teledyne Hanson, Chatsworth, CA, USA) according to the conditions specified in the individual USP monographs for each drug, which are summarized in Table S1, Supporting Information. Samples (1 mL) were withdrawn at specified time points and centrifuged at 3000 g prior to quantification according to the analytical procedures described in the Instrumentation and Analytical Methods section. Experiments were performed in quadruplicates.

### Cost Effectiveness Analysis

A polypill cost effectiveness analysis for a cardiovascular disease application was conducted using a Markov model to compare the VD to a FDC polypill strategy and a standard care strategy using TreeAge Pro Healthcare 2020 (Williamstown, Massachusetts). The base case cohort followed was 60‐year‐old men and women with a defined cardiovascular risk but no prior cardiovascular event such as MI. This risk was defined as having one or more of the following CVD risk factors: >160 mg dl^−1^ systolic BP, obesity, hyperlipidemia, a diagnosis of coronary heart disease, or having a Framingham 5‐year CVD risk value of greater than 7.5%). The above criteria were chosen since these were among the most common seen inclusion criteria included in the clinical trials used to support the model.^[^ ^20^, ^35^, ^36^, ^37^, ^38^, ^39^ ^]^ This cohort was followed over a lifetime time horizon with a Markov cycle length of 1 year. The health states through which the cohort went through is shown in Figure 5A. Variables in the analysis included medication adherence, side effects, discontinuation of the medication, and possibility of MI. Probabilities, utilities, and costs were taken from existing literature^[^ ^19^, ^20^, ^27^, ^35^, ^36^, ^37^, ^38^, ^39^, ^40^, ^41^, ^42^ ^]^ and used as model inputs for the base case analysis. All probabilities used are listed in Table S2, Supporting Information and all utilities and costs used are listed in Table S3, Supporting Information. The primary outcome was the ICER per QALY between competing treatment strategies. The willingness‐to‐pay threshold was $100 000/QALY as in other studies.^[^ ^43^ ^]^ Costs were converted to 2019 USD using the medical Consumer Price Index. 3% discounting was applied to all costs used within the model.

### Statistical Analysis

Experimental results are presented as mean values ± standard deviations (SD). Statistical analysis was performed using OriginPro 9.0 applying one‐way analysis of variance and statistical significance was set at p<0.05.

## Conflict of Interest

The authors declare no conflict of interest.

## Supporting information

Supporting Information

## Data Availability

The data that support the findings of this study are available from the corresponding author upon reasonable request.
