# Supplementary material for: Modular Titratable Polypills for Personalized Medicine and Simplification of Complex Medication Regimens
Source: Adv Healthc Mater. 2023 Aug 13;12(27):2301101. doi: 10.1002/adhm.202301101 (PMC10836191; doi:10.1002/adhm.202301101)
Supplement: Supplementary file 1 — Supporting Information [file ADHM-12-2301101-s001.pdf]

ADVANCED HEALTHCARE MATERIALS

Supporting Information

for *Adv. Healthcare Mater.*, DOI 10.1002/adhm.202301101

Modular Titratable Polypills for Personalized Medicine and Simplification of Complex Medication Regimens

*Christina Karavasili, Sahab Babaee, Shruti Kutty, Jacqueline N. Chu, Seokkee Min, Nina Fitzgerald, Joshua Morimoto, Nicoletta Inverardi and Giovanni Traverso**

Supporting Information

Modular Titratable Polypills for Personalized Medicine and Simplification of Complex Medication Regimens

*Christina Karavasili, Sahab Babaee, Shruti Kutty, Jacqueline N. Chu, Seokkee Min, Nina Fitzgerald, Joshua Morimoto, Nicoletta Inverardi, Giovanni Traverso**

C. Karavasili, S. Babaee, S. Kutty, J.N. Chu, S. Min, N. Fitzgerald, J. Morimoto, N. Inverardi, G. Traverso
David H. Koch Institute for Integrative Cancer Research and Department of Chemical Engineering, Massachusetts Institute of Technology, Cambridge, MA, 02139, USA

C. Karavasili, S. Babaee, J.N. Chu, G. Traverso
Division of Gastroenterology, Brigham and Women's Hospital, Harvard Medical School, Boston, MA 02115, USA

J.N. Chu
Integrated Gastroenterology Consultants, N. Chelmsford, MA, 01863, USA

S. Babaee, S. Min, G. Traverso
Department of Mechanical Engineering, Massachusetts Institute of Technology, Cambridge, MA 02139, USA.

*E-mail: cgt20@mit.edu

Table S1. *In vitro* dissolution test conditions and acceptance criteria as specified in the respective USP drug monographs (USP29-NF24).

Drug	Medium	Medium volume	Rotational speed	Apparatus	Tolerances
**Rosuvastatin**	Citrate buffer pH 6.6	900 mL	50 rpm	2	Not less than 75% dissolved in 30 minutes
**Lisinopril**	0.1 N hydrochloric acid	900 mL	50 rpm	2	Not less than 80% dissolved in 30 minutes
**Hydrochlorothiazide**	0.1 N hydrochloric acid	900 mL	100 rpm	1	Not less than 60% dissolved in 60 minutes
**Acetyl salicylic acid**	0.05 M acetate buffer pH 4.5	500 mL	50 rpm	1	Not less than 80% dissolved in 30 minutes
**Isoniazid**	0.01 N hydrochloric acid	900 mL	100 rpm	1	Not less than 80% dissolved in 45 minutes
**Rifampicin***	0.1 N hydrochloric acid	900 mL	100 rpm	1	Not less than 75% dissolved in 45 minutes
**Ethambutol**	Water	900 mL	100 rpm	1	Not less than 75% dissolved in 45 minutes
**Pyrazinamide**	Water	900 mL	50 rpm	2	Not less than 75% dissolved in 45 minutes
**Prednisone**	Water (vacuum filtration at 41 °C, then cooling to 37 °C before use)	500 mL	50 rpm	2	Not less than 80% dissolved in 30 minutes

***** The conditions specified in the USP monograph of rifampicin capsules were applied for the dissolution testing of the rifampicin modular tablets.

Table S2. Model inputs for probabilities for the cost-effectiveness model.

Parameter	Base Case Value	Range for Sensitivity Analysis
Age	60 [1–5]	40[1] - 80[1] (estimate + 5 years)
Survival for patients who have had an event		
Year 1	0.845[6]	0.845[6] – 0.96[7]
Year 2	0.98[6]	0.945[7] – 0.98[6]
Year 3 and onwards	0.985[6]	0.965[7] – 0.985[6]
**Probabilities (annual)**		
**Standard Care**		
Adherence	0.65[8]	0.46[9] – 0.997[10]
Side effects	0.4026[10]	0.01566[4,5] – 0.8651[3]
Discontinuation after having side effects	0.0788[10]	0.04773[4] – 0.118215[10] (Estimate +50%)
Continue treatment and have event	0.01825[1]	0.002998[7] – 0.027375[1] (Estimate +50%)
Stop treatment after side effects and have event	0.01903[1]	0.0093312416[4,5] – 0.06295[7]
No side effects and have event	0.01825[1]	0.002998[7] – 0.027375[1] (Estimate +50%)
Low adherence to treatment and have event	0.06295[7]	0.044491585[7] – 0.155533[7]
Well off treatment and have event	0.06295[7]	0.044491585[7] – 0.155533[7]
**FDC**		
Adherence	0.88[8]	0.81[9] – 0.994[10]
Side effects	0.62985[10]	0.45415[10] (Estimate*) – 0.8883[3]
Discontinuation after having side effects	0.18549[10]	0.06126[8] – 0.57[9]
Continue treatment and have event	0.00813[1]	0.002690625[1] – 0.0253[8]
Stop treatment after side effects and have event	0.01903[1]	0.0093312146[4] – 0.06295[7]
No side effects and have event	0.00813[1]	0.002690625[1] – 0.0253[8]
Low adherence to treatment and have event	0.0189[1]	0.016276632[1] – 0.02543[1]
Well off treatment and have event	0.0189[1]	0.016276632[1] – 0.02543[1]
**VD**		

Adherence	0.88[8]	0.81[9]– 0.994[10]
Side effects	0.4026[10]	0. 0.01566[4,5] – 0.8651[3]
Discontinuation after having side effects	0.0788[10]	0.04773[4] – 0.118215[10] (Estimate +50%)
Continue treatment and have event	0.00813[1]	0.002690625[1] – 0.0154084[8]
Stop treatment after side effects and have event	0.01903[1]	0.0093312146[4] – 0.06295[7]
No side effects and have event	0.00813[1]	0.002690625[1] – 0.0139953[8]
Low adherence to treatment and have event	0.0189[1]	0.016276632[1] – 0.02543[1]
Well off treatment and have event	0.0189[1]	0.016276632[1] – 0.02543[1]

*Values estimated from base case values from reference papers.

Table S3. Model inputs for utility and cost values for the cost-effectiveness model. Costs are presented in 2019 US$. Red text indicates health states associated with each utility.

**Parameter**	**Base Case value**	**Range for Sensitivity Analysis**
**Utilities**		
CVD risk (Well on/off Rx)	0.95[11]	0.85[12] – 0.989[11]
Prior CVD event (Post event)	0.79[13]	0.70[14] – 0.88[15]
Polypharmacy (Post event)	0.69[16]	0.68[16] – 0.71[16]
Disutility for Side effects (Myalgia)	-0.017[17]	-0.0829[18] – -0.123[19]
Disutility for MI	-0.24[20]	-0.125[21] – -0.36[13]
**Costs**		
Lisinopril (yearly)	$57.84[22]	$43.38* – $72.30*
Aspirin (yearly)	$14.28[23]	$10.71* - $17.85*
Hydrochlorothiazide (yearly)	$37.08[24]	$27.81* - $46.35*
Rosuvastatin (yearly)	$101.88[25]	$76.41* - $127.35*
Doctor's visit	$139.05/visit[26]	$81.51[15] - $173.81*
Pharmacist salary/hour	$61.58[27]	$46.18* - $76.98*
MI hospitalization	$26,573.16[28]	$18,665.23[28] - $32,304.22[28]
Polypill cost (including pharmacist)	$41.40/month[2]	$12** - $200**

*Costs were obtained by estimating +/- 25% from the base case value.
**Costs estimated to test a large range of values due to lack of availability of polypill cost data in the literature.

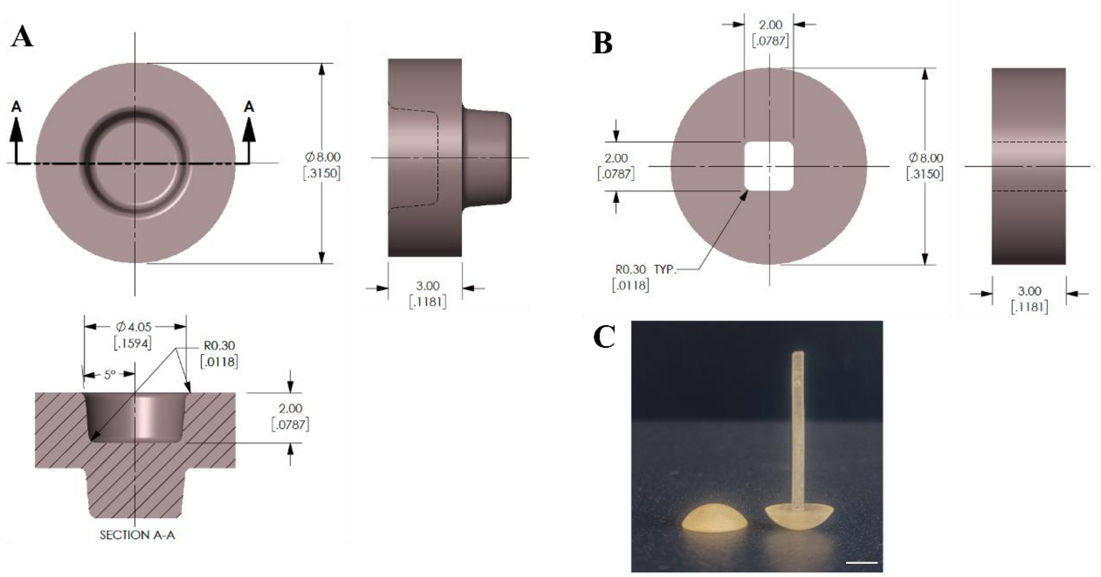

Figure S1. Detailed CAD models of the polypill prototypes at different views for Design 1 (A) and Design 2 (B). The dimensions are in mm [in]. (C) The isomaltose-casted dissolvable parts used for the assembly of the annular disk-shaped tablets (Design 2). Scale bar corresponds to 0.5 cm.

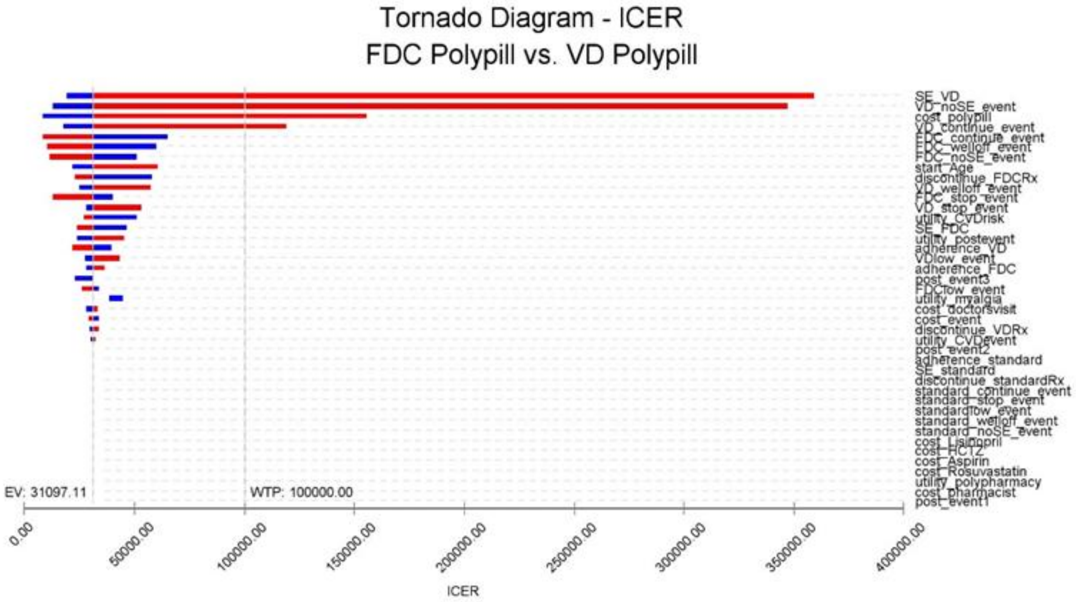

Figure S2. Tornado diagram of one-way sensitivity analyses for all of the parameters in the model. Variation of only four parameters changed the preferred strategy (ICER crossed the willingness-to-pay threshold of $100,000/QALY): VD side effects, probability of having an event with the VD strategy, and cost of the VD polypill.

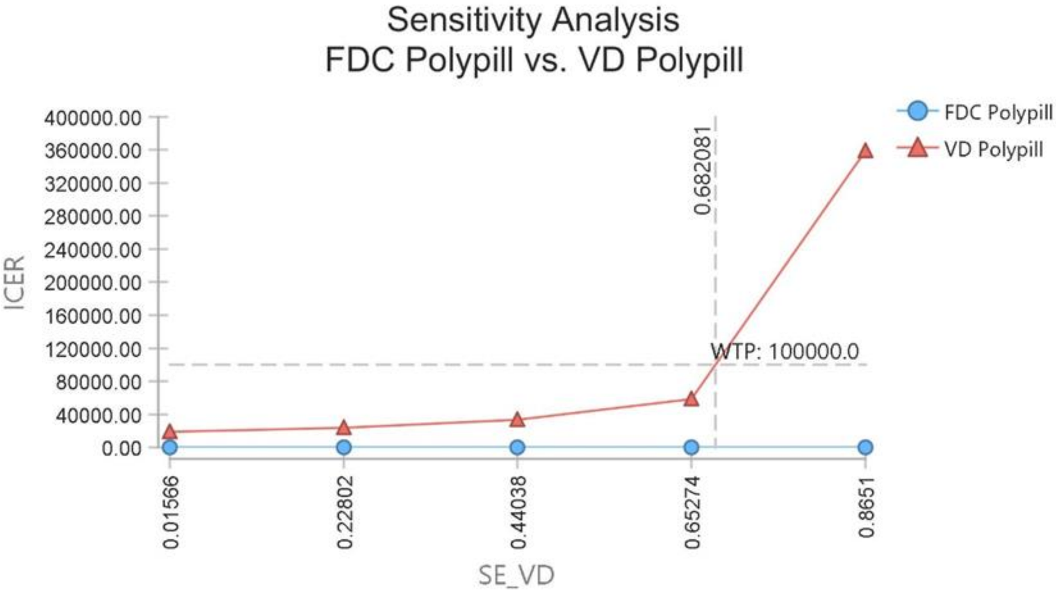

Figure S3. One-way sensitivity analysis of probability of side effects for VD patients. Beyond 68% side effects yearly for patients, the VD polypill stops being cost effective.

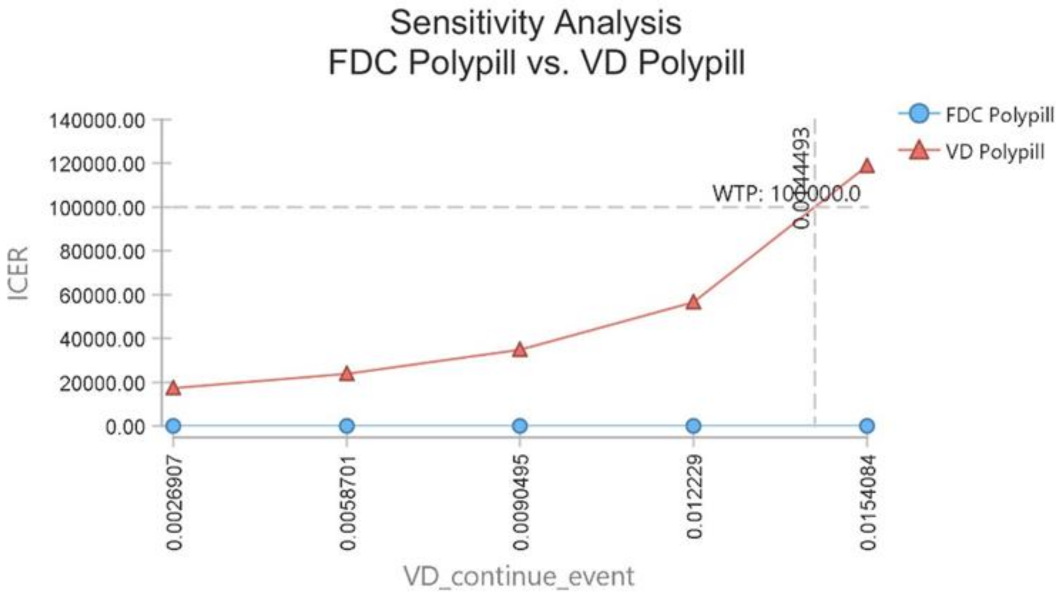

Figure S4. One way sensitivity analysis for the probability of VD patients who experience an event while experiencing side effects and continuing their medication. If this probability exceeds beyond 1.4%, the VD polypill stops being cost effective.

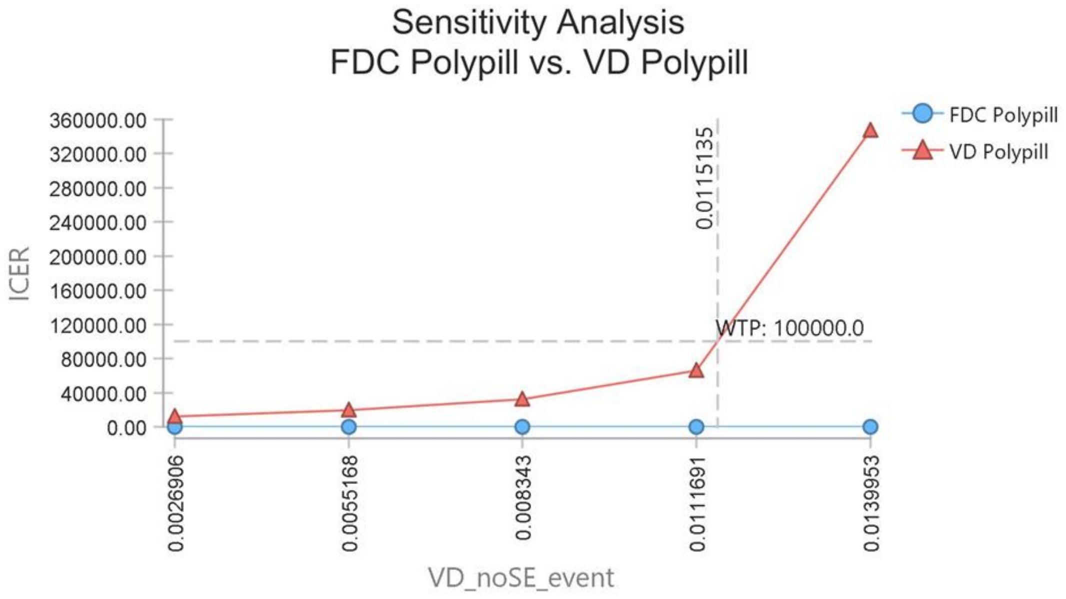

Figure S5. One way sensitivity analysis for the probability of VD patients experiencing an event while experiencing no side effects from the medication. If this exceeds 1.1%, the VD polypill stops being cost effective.

References

[1] G. Roshandel, M. Khoshnia, H. Poustchi, K. Hemming, F. Kamangar, A. Gharavi, M. R. Ostovaneh, A. Nateghi, M. Majed, B. Navabakhsh, S. Merat, A. Pourshams, M. Nalini, F. Malekzadeh, M. Sadeghi, N. Mohammadifard, N. Sarrafzadegan, M. Naemi-Tabiei, A. Fazel, P. Brennan, A. Etemadi, P. Boffetta, N. Thomas, T. Marshall, K. K. Cheng, R. Malekzadeh, *Lancet* **2019**, *394,* 672.

[2] D. Muñoz, P. Uzoije, C. Reynolds, R. Miller, D. Walkley, S. Pappalardo, P. Tousey, H. Munro, H. Gonzales, W. Song, C. White, W. J. Blot, T. J. Wang, *N. Engl. J. Med.* **2019**, *381*, 1114.

[3] E. Z. Soliman, S. Mendis, W. P. Dissanayake, N. P. Somasundaram, P. S. Gunaratne, I. K. Jayasingne, C. D. Furberg, *Trials* **2011**, *12*, 3.

[4] E. M. Lonn, J. Bosch, P. López-Jaramillo, J. Zhu, L. Liu, P. Pais, R. Diaz, D. Xavier, K. Sliwa, A. Dans, A. Avezum, L. S. Piegas, K. Keltai, M. Keltai, I. Chazova, R. J. G. Peters, C. Held, K. Yusoff, B. S. Lewis, P. Jansky, A. Parkhomenko, K. Khunti, W. D. Toff, C. M. Reid, J. Varigos, L. A. Leiter, D. I. Molina, R. McKelvie, J. Pogue, J. Wilkinson, H. Jung, G. Dagenais, S. Yusuf, *N. Engl. J. Med.* **2016**, *374*, 2009.

[5] S. Yusuf, J. Bosch, G. Dagenais, J. Zhu, D. Xavier, L. Liu, P. Pais, P. López-Jaramillo, L. A. Leiter, A. Dans, A. Avezum, L. S. Piegas, A. Parkhomenko, K. Keltai, M. Keltai, K. Sliwa, R. J. G. Peters, C. Held, I. Chazova, K. Yusoff, B. S. Lewis, P. Jansky, K. Khunti, W. D. Toff, C. M. Reid, J. Varigos, G. Sanchez-Vallejo, R. McKelvie, J. Pogue, H. Jung, P. Gao, R. Diaz, E. Lonn, *N. Engl. J. Med.* **2016**, *374,* 2021.

[6] R. Radisauskas, J. Kirvaitiene, G. Bernotiene, D. Virviciutė, R. Ustinaviciene, A. Tamosiunas, *Medicina (Kaunas).* **2019**, *55*, 357.

[7] A. K. Gehi, S. Ali, B. Na, M. A. Whooley, *Arch. Intern. Med.* **2007**, *167,* 1798.

[8] S. Thom, N. Poulter, J. Field, A. Patel, D. Prabhakaran, A. Stanton, D. E. Grobbee, M. L. Bots, K. S. Reddy, R. Cidambi, S. Bompoint, L. Billot, A. Rodgers, for the U. C. Group, *JAMA* **2013**, *310*, 918.

[9] V. Selak, C. R. Elley, C. Bullen, S. Crengle, A. Wadham, N. Rafter, V. Parag, M. Harwood, R. N. Doughty, B. Arroll, R. J. Milne, D. Bramley, L. Bryant, R. Jackson, A. Rodgers, *BMJ Br. Med. J.* **2014**, *348,* g3318.

[10] P. C. Group, A. Rodgers, A. Patel, O. Berwanger, M. Bots, R. Grimm, D. E. Grobbee,

R. Jackson, B. Neal, J. Neaton, N. Poulter, N. Rafter, P. K. Raju, S. Reddy, S. Thom, S. Vander Hoorn, R. Webster, *PLoS One* **2011**, *6*, e19857.

[11] B. S. Ferket, M. G. M. Hunink, M. Khanji, I. Agarwal, K. E. Fleischmann, S. E. Petersen, *Heart* **2017**, *103*, 483 LP.

[12] R. Kumar, A. Tonkin, D. Liew, E. Zomer, *Int. J. Cardiol.* **2018**, *267*, 183.

[13] K. Kongpakwattana, Z. Ademi, T. Chaiyasothi, S. Nathisuwan, E. Zomer, D. Liew, N. Chaiyakunapruk, *Pharmacoeconomics* **2019**, *37*, 1277.

[14] S. Jowett, P. Barton, A. Roalfe, K. Fletcher, F. D. R. Hobbs, R. J. McManus, J. Mant, *PLoS One* **2017**, *12*, e0182625.

[15] J. F. Slejko, R. L. Page, P. W. Sullivan, *Curr. Med. Res. Opin.* **2010**, *26*, 2485.

[16] F. Jódar-Sánchez, A. Malet-Larrea, J. J. Martín, L. García-Mochón, M. P. López del Amo, F. Martínez-Martínez, M. A. Gastelurrutia-Garralda, V. García-Cárdenas, D. Sabater-Hernández, L. Sáez-Benito, S. I. Benrimoj, *Pharmacoeconomics* **2015**, *33*, 599.

[17] J. Jarmul, M. J. Pletcher, K. Hassmiller Lich, S. B. Wheeler, M. Weinberger, C. L. Avery, D. E. Jonas, S. Earnshaw, M. Pignone, *Circ. Cardiovasc. Qual. Outcomes* **2018**, *11*, e004171.

[18] D. Mitchell, J. R. Guertin, A. C. Iliza, F. Fanton-Aita, J. LeLorier, *Mol. Diagn. Ther.* **2017**, *21*, 95.

[19] Y. Hagiwara, T. Shiroiwa, K. Shimozuma, T. Kawahara, Y. Uemura, T. Watanabe, N. Taira, T. Fukuda, Y. Ohashi, H. Mukai, *Pharmacoeconomics* **2018**, *36*, 215.

[20] S. K. Gandhi, M. M. Jensen, K. M. Fox, L. Smolen, A. G. Olsson, T. Paulsson, *Clinicoecon. Outcomes Res.* **2012**, *4*, 1.

[21] J. Salcedo, J. W. Hay, J. Lam, *Int. J. Cardiol.* **2019**, *282*, 53.

[22] Goodrx.com (2019)., .

[23] GoodRx.com (2019). Retrieved from https://www.goodrx.com/aspirin, .

[24] GoodRx.com (2019). Retrieved from https://www.goodrx.com/hydrochlorothiazide, .

[25] GoodRx.com (2019). Retrieved from https://www.goodrx.com/rosuvastatin?dosage=5mg&form=tablet&label_override=rosuvastatin&quantity=30, .

[26] data.cms.gov (10 August 2019). Retrieved from: https://data.cms.gov/provider-summary-by-type-of-service/medicare-physician-other-practitioners/medicare-physician-other-practitioners-by-geography-and-service/data/2017, .

[27] Bls.org (2019). Retrieved from https://www.bls.gov/ooh/healthcare/pharmacists.htm#:~:text=%2439%2C810-,The%20median%20annual%20wage%20for%20pharmacists%20was%20%24128%2C090%20in%20May,percent%20earned%20more%20than%20%24162%2C900, .

[28] G. Nicholson, S. R. Gandra, R. J. Halbert, A. Richhariya, R. J. Nordyke, *Clinicoecon. Outcomes Res.* **2016**, *8*, 495.
